# The influence of psychological interventions on surgical outcomes: a systematic review

**DOI:** 10.1186/s44158-022-00057-4

**Published:** 2022-07-09

**Authors:** Iacopo Lanini, Timothy Amass, Caterina Scirè Calabrisotto, Sergio Fabbri, Silvia Falsini, Chiara Adembri, Alessandro Di Filippo, Stefano Romagnoli, Gianluca Villa

**Affiliations:** 1 Department of Health Sciences, Section of Anesthesiology, and Intensive Care, University of Florence, Florence, Italy; 2 Department of Medicine, Division of Pulmonary Sciences and Critical Care Medicine, University of Colorado, Denver, CO USA; 3 Department of Anesthesia and Intensive Care, Azienda Ospedaliero Universitaria Careggi, Florence, Italy

**Keywords:** Metabolic stress response, Perioperative care, Cognitive behavior therapy, Mindfulness, Narrative medicine

## Abstract

**Background:**

An amplified and/or prolonged surgical stress response might overcome the organs’ functional reserve, thus leading to postoperative complications. The aim of this systematic literature review is to underline how specific psychological interventions may contribute to improve surgical outcomes through the positive modulation of the surgical stress response in surgical patients.

**Methods:**

We conducted a comprehensive literature search in the Cochrane Register of Controlled Trials, PubMed, EMBASE, Scopus, PsycINFO, and CINAHL databases. Only studies published in English from Jan 2000 to Apr 2022 and reporting pain and/or anxiety among outcome measures were included in the review. The following psychological interventions were considered: (1) relaxation techniques, (2) cognitive-behavioral therapies, (3) mindfulness, (4) narrative medicine, (5) hypnosis, and (6) coping strategies.

**Results:**

Among 3167 records identified in the literature, 5 papers were considered eligible for inclusion in this review because reporting the effects that psychological features have on neurochemical signaling during perioperative metabolic adaptation and those metabolic and clinical effects that the psychological interventions had on the observed population.

**Conclusion:**

Our findings confirm that psychological interventions may contribute to improve surgical outcomes via the positive influence on patients’ metabolic surgical stress response. A multidisciplinary approach integrating physical and non-physical therapies can be considered a good strategy to successfully improve surgical outcomes in the perioperative period.

**Supplementary Information:**

The online version contains supplementary material available at 10.1186/s44158-022-00057-4.

## Background

Postoperative complications and undesirable sequelae of surgery (such as pain, fatigue, depression, and prolonged convalescence) occur frequently, particularly in frail patients [1]. Their development is usually associated with a maladaptive response to surgical stress, a condition that includes alterations in metabolic and physiologic processes that induce perturbations in inflammatory, acute-phase, hormonal, and genomic responses [2]. The result of hypermetabolism and hypercatabolism leads to muscle wasting, impaired immune function and wound healing, organ failure, and death [3].

In some patients, surgical stress is amplified and/or prolonged to such extent as to overcome the organs’ functional reserve [4]. Under these conditions, body mass anabolism, inflammation, tissue regeneration, immunological system, and organ function recovery are impaired [4] and can lead to worsened patients’ outcomes.

Several perioperative variables (e.g., blended anesthesia, opioids sparing strategies, or minimally invasive surgery) seem to counteract the pathophysiological mechanisms behind maladaptive surgical stress response. Nonetheless, no single anesthesiologic or surgical option has been demonstrated to be able to completely eliminate postoperative morbidity and mortality [5, 6]. In this context, specific psychological interventions aimed at preventing maladaptive psychological features have been demonstrated to be effective in modulating the surgical stress response in surgical patients [7]. For this reason, the implementation of this kind of intervention is recommended in perioperative care.

In this paper, we have introduced issues and research that have been identified in psychology as relevant to surgical care promoting the perioperative integration of physical and non-physical treatments aimed at modulating the surgical stress response. In particular, we have systematically reviewed the literature reporting the repercussions that psychological features have on neuro-chemical signaling during perioperative metabolic adaptation and the clinical effects that the most common psychological interventions have on surgical patients when approaches such as cognitive-behavioral interventions and narrative medicine are applied perioperatively.

## Methods

All authors performed a comprehensive literature search in the following electronic databases: the Cochrane Register of Controlled Trials, PubMed, EMBASE, Scopus, PsycINFO, and CINAHL. This systematic review was developed according to the PRISMA methodology [8]. The protocol for this systematic review was not registered. The following search terms were used: “surgery,” “cognitive behavioral therapy,” “relaxation therapy,” “mindfulness,” “coping,” “hypnosis,” “narrative medicine,” “psychological intervention,” “pain,” and “anxiety.” The research has been limited to the papers published in English from Jan 2000 to April 2022. The complete search strategy for each database is provided as supplementary material. The eligible studies, identified following the PICO criteria, were (1) patients undergoing elective or emergency surgery; (2) psychological interventions as relaxation techniques, cognitive-behavioral therapies, mindfulness, narrative medicine, hypnosis, and coping strategies; and (3) outcome as pain and anxiety. Two independent reviewers (S.F. and G.V.) assessed the identified papers. Titles and abstracts were evaluated for eligibility criteria; papers primarily focused on the pediatric population or intraoperative awareness were excluded in this phase. Conflicts and disagreements were discussed with all other authors, and a unified list of eligible papers was defined. The full text of eligible papers was analyzed, and studies not reporting explicitly the psychological interventions were excluded, as well as those not reporting the effects that psychological features had on neurochemical signaling during perioperative metabolic adaptation, and those metabolic and clinical effects that the psychological interventions had on the observed population. Commentaries, letters, editorials, case reports, case series, reviews, and meta-analysis were not included for the final analysis.

## Results

**Fig. 1:**
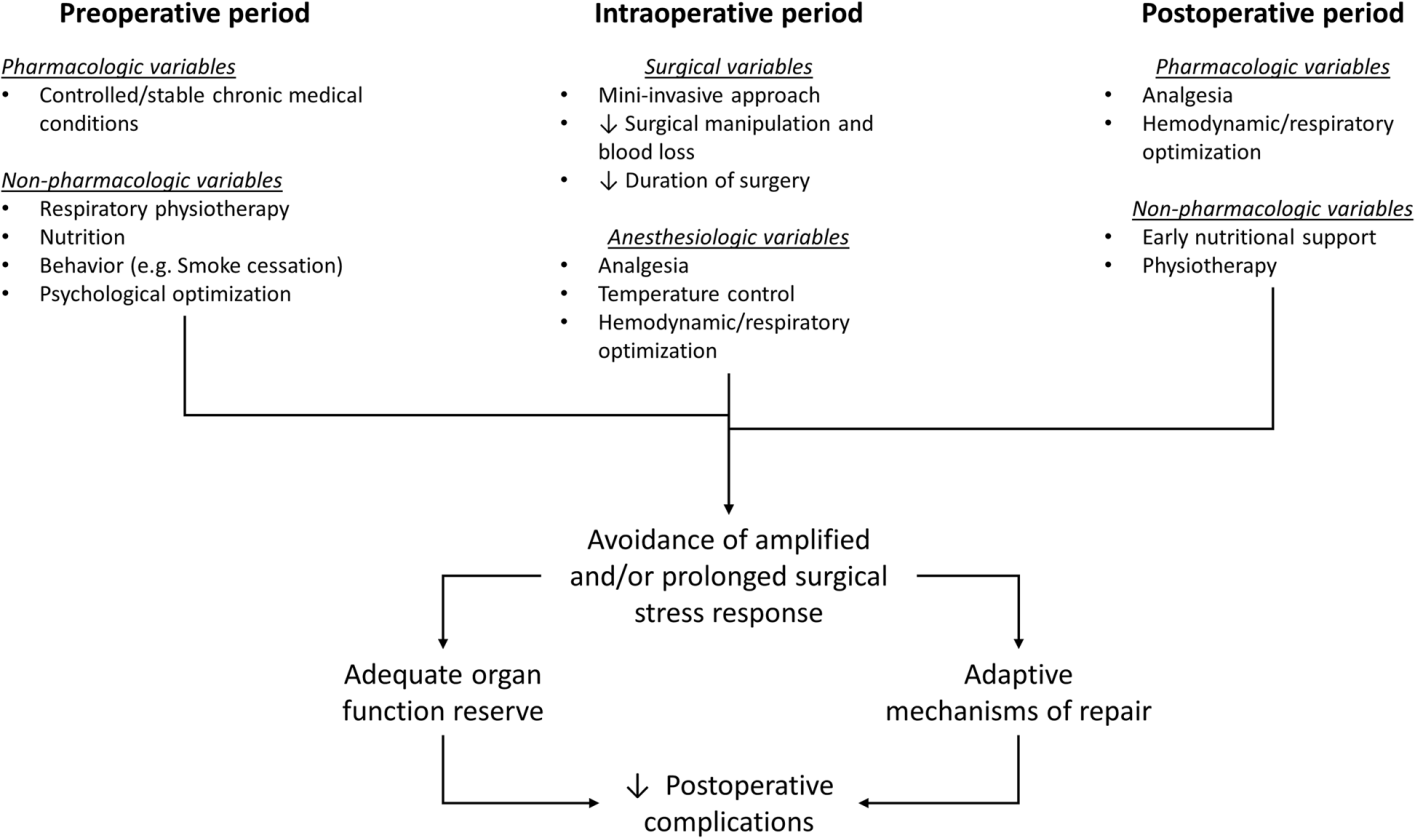
Study selection process

Three thousand one hundred and sixty-seven records were extracted from the literature. Figure 1 shows the selection process. After duplicates removal, title and abstracts of 1934 papers were screened and 1745 of them were excluded. The full text was analyzed for 189 papers in the elegibility phase, and only 5 papers were considered for discussion in this review (Table 1).

**Table 1:** Papers selected and considered for discussion

	Surgical population	Psychological interventions	Pathophysiologic marker	Surgical outcomes
Rosenberger et al. 2004	Knee arthroscopic surgery	Coping strategies	Preoperative vs 1 week after surgery	Preoperative vs 24 weeks after surgery
			*Cortisol levels (ug/dL):* Pts Active cooping 27 ± 2 vs 25 ± 2 Pts Avoidant cooping 22 ± 2 vs 20 ± 2	*West Haven Yale Multidimensional Pain Inventory* Pts Active cooping 1.8 ± 0.2 vs 0.9 ± 0.2 Pts Avoidant cooping 1.8 ± 0.2 vs 1.2 ± 0.2
			*P < 0.05 overtime reduction with ANCOVA analysis.	*P < 0.05 overtime reduction with ANCOVA analysis.
Thornton et al. 2009	Breast cancer	Relaxation techniques and coping strategies	Effect estimates of Psyc. Interventions on metabolic variables [estimate (SE)]:	Effect estimates of Psyc. Interventions on surgical outcomes [estimate (SE)]:
			*White blood cells count:* 0.45 (0.49) *Neutrophil count:* 0.16 (0.37) *T-helper count:* 0.59 (0.34)	*Bodily Pain subscale* from the Medical Outcomes Study-Short form (SF-36): −8.42 (6.86)
Zhao et al. 2016	Lung resection	Relaxation techniques	Pre vs post intervention	Preoperative vs 24 h after surgery
			*CD3*^*+*^*levels (%):* CG: 65.4 ± 11.3 vs 69.3 ± 11.1 PsycG: 64.2 ± 9.4* vs 67.2 ± 8.8	*VAS* CG: 5.4 ± 1.3 vs 7.3 ± 1.3 PsycG: 2.7 ± 1.9* vs 4.2 ± 1.4*
			*CD4*^*+*^*levels (%):* CG: 39.2 ± 7.6 vs 4.1 ± 10.0 PsycG: 35.8 ± 7.9* vs 39.9 ± 8.1**	
			*Cortisol levels (nmol/L):* CG: 494.7 ± 153.6 vs 471.3 ± 91.7 PsycG: 576.7 ± 125.1* vs 507.9 ± 131.9	
			*P < 0.05 in comparison between experimental group and control group. **P < 0.05 in comparison before and after intervention in experimental group.	*P* < 0.05 in comparison between experimental group and control group.
Marques dos Santos Felix et al. 2018	Video-laparoscopic bariatric surgery	Relaxation therapy with guided imagery	Pre vs post intervention	Pre vs post intervention
			*Cortisol levels (ug/dL):* CG: 8.6 ± 5.4 vs 8.2 ± 5.3 PsycG: 9.9 ± 6.1 vs 7.9 ± 5.4*	*State-Trait Anxiety Inventory scale* CG: 47.7 ± 3.8 vs 46.8 ± 3.7 PsycG: 47.5 ± 2.6 vs 43.0 ± 3.5*
			*P < 0.001 in comparison before and after intervention in the experimental group.	*P < 0.001 in comparison before and after intervention in experimental group.
Mirmahmoodi et al. 2020	Breast cancer	Mindfulness	Pre vs post intervention	Pre vs post intervention
			*Cortisol levels (ug/dL):* CG: 16.5 ± 3.8 vs 16.7 ± 4.0* PsycG: 18.8 ± 4.1 vs 18.3 ± 4.3*	*Anxiety score:* CG: 25.4 ± 15.0 vs 35.0 ± 13.5* PsycG: 31.18 ± 14.05 vs 23.50** ± 11.35*
			*CRP levels (mg/dL):* CG: 12.0 ± 6.2 vs 11.5 ± 6.3 PsycG: 15.5 ± 5.2 vs 14.3 ± 5.3	
			*P < 0.05 in comparison before and after intervention in both groups.	*P < 0.05 in comparison before and after intervention in both groups. **P < 0.05 in comparison between the experimental group and control group.

## Discussion

Basing on the current understanding of the pathophysiological mechanisms of maladaptive surgical stress response, this systematic review describes the repercussions that psychological interventions have on neuro-chemical signaling during perioperative metabolic adaptation, and the clinical effects that the most common psychological interventions have on surgical patients.

### Pathophysiological mechanisms of maladaptive surgical stress response

Stress is defined as a “specific response by the body to a stimulus, as fear or pain, that disturbs or interferes with the normal physiological equilibrium of an organism.” It can be “external” (induced by environmental factors and psychological or social situations) or “internal” (due to illness or iatrogenesis, that is, resulting from a medical procedure). Accordingly, psychological/biochemical stress in the perioperative period can derive from environmental stressors as well as from surgical aggression. Stress can trigger or influence the course of many medical conditions, including psychological disorders (e.g., depression and anxiety) or organic diseases (e.g., perioperative outcomes). It induces a standardized, non-species-specific, well-organized, and predictable response, which is adaptive in nature and aimed at providing an adequate amount of energy substrate and amino acids for the synthesis of visceral proteins and the organism’s healing process [4].

A physiological, balanced, and well-controlled response usually results in complete and rapid recovery from the surgical procedure [2]. However, pre-existing diseases as well as the patient’s genetic predisposition may induce a dysfunctional adaptation leading to an exaggerated inflammation (i.e. systemic inflammatory response syndrome, SIRS) or to an inadequate response (i.e., anergy) [4].

The metabolic surgical stress response is thus a functional adaptation which occurs in surgical patients and is sustained by the activation of trauma-induced neuroendocrine pathways and several inflammatory mediators (e.g., cytokines, complement, arachidonic acid metabolites, nitric oxide, and free oxygen radicals) [3]. In particular, the neuroendocrine response is characterized by an increased secretion of epinephrine and cortisol, as well as of glucagon, growth hormone, aldosterone, and arginine-vasopressin. Hemodynamic and metabolic variations during surgical stress events elicit a prompt secretion of epinephrine induced by activation of the sympathetic nervous system. Furthermore, afferent impulses from the damaged tissue stimulate the secretion of the hypothalamic releasing hormone with amplitude and duration correlated with the extent of the surgical trauma [4, 10]. Beyond the hypothalamic-pituitary-adrenal axis, the endocrine response to surgical stress involves several hormonal axes; it is well organized, self-limited, and mainly promotes the metabolic adaptation during surgical stress [4, 10].

Inflammatory mediators, and mainly cytokine response, during stress events have been extensively studied. Specifically, cytokines response is usually characterized by production and release of a wide range of pro-inflammatory mediators and their physiologic modulating compensatory substances, i.e., anti-inflammatory mediators. The co-expression of inflammatory and anti-inflammatory pathways, as well as the controlled predominance of their mediators with a specific timing after the surgical insult, guarantee an adaptive inflammatory response and avoid disorders leading to SIRS or anergy. Furthermore, the release of cytokines regulates the immuno-inflammatory response promptly initiated after the acute stressor event. Indeed, cytokines promote communication among leukocytes by linking innate and adaptive immune responses [11, 12].

The wide interaction between neuroendocrine and cytokine mediators has also been demonstrated to influence the regulation of the metabolic stress response [10]. For example, tumor necrosis factor (TNF)-a, interleukin (IL)-1, and IL-6 induce the activation of the hypothalamic-pituitary-adrenal axis [13]. In healthy subjects, the administration of TNF-a induces high plasma levels of corticotropin, cortisol, catecholamines, growth hormone, and glucagon, that is, a hormonal response comparable to the one observed during stress events [14]. Corticotropin-releasing hormone, which is released by the hypothalamus during the stressor event, is also produced by leukocytes [15]. Finally, immune cells are also recognized as a new and widely distributed adrenergic organ which generates and releases catecholamines [16].

These pathophysiological phenomena have been explored in detail in this review and represent a novelty compared to similar reviews carried out on different populations such as that conducted by Villa G. et al. in 2020, carried out on an abdominal surgery population [2]. It is worth noting that also psychological, perioperative patient factors (e.g., psychological state and/or personality) may directly affect the surgical stress response according to different mechanisms [17]. Interestingly, some studies have shown that these factors can predict postoperative outcomes with more accuracy than surgical or anesthesiologic variables [18].

### Psychological features and perioperative metabolic adaptation

Negative psychological states, and their indirect influences on patient behavior (e.g., obesity, smoking, alcohol intake), may affect surgical recovery [17, 19]. In particular, patients’ psychological features may directly interact with the neuroendocrine and inflammatory pathways underlying the surgical stress response [17], with major repercussions on immunological perioperative state and surgical outcomes [20].

The activation of the autonomic nervous system during an acute stressor event may induce the sympathetic fibers to release a wide range of mediators, directly affecting the immune response [21]. Furthermore, the density and sensitivity of adrenergic receptors to different components of the immune system may affect the responsiveness of cell subsets to stressor events [22]. Similarly, the several hormones released through the stress-induced activation of the hypothalamic-pituitary-adrenal axis (e.g., the adrenal hormones epinephrine, norepinephrine, cortisol, prolactin, growth hormone, and the brain peptides melatonin, β-endorphin, and enkephalin) may influence the immune response. In particular, these hormones may bind specific receptors to the immune cells and regulate their distribution and function [21]. As a consequence, different patterns of activation may be recognized during stressor events, with potentially adaptive upregulation of the natural immunity and downregulation of the specific immunity [22]. In a meta-analysis considering more than 300 papers, Segerstrom et al. described the pathophysiological relationship between hormonal alteration during psychological stress and the immune system [22].

Interactions between psychological factors and inflammation (expressed as cytokine modulation) are well established in the literature [23], also in surgical settings [24]. These interactions may lead to maladaptive mechanisms of perioperative inflammation and play a role in the development of postoperative complications. Like the neuroendocrine response, the cytokine response to surgical stress may also influence the immune function. Several studies have reported correlations between psychological stress and reduced natural killer cell cytotoxicity, suppressed lymphocyte proliferation, and blunted humoral responses to immunization [22, 25]. An ineffective immune response is considered the main cause of the high incidence of infections among chronically stressed individuals [22]. Finally, a pathological pattern of cytokine secretion has been recognized during stressor events that may lead to an imbalance between cellular Th-1 and humoral Th-2 activation and, as a consequence, to infectious/autoimmune diseases [22].

Significantly, the psychoneuroendocrine characteristics, and more in general the psychosomatic effects of psychological stress on inflammation and surgical response, might be modulated by psychological treatments. Most psychological therapies are associated with increased secretion of inhibiting hypothalamic hormones, such as somatostatin or dopamine, and decreased secretion of releasing hormones, such as thyrotropin- and corticotropin-releasing hormones and the growth hormone-releasing factor. Thus, cortisol levels decrease whereas levels of beta-endorphins may increase [26]. A similar restoration of physiological neuroendocrine adaptation is also reported. All these effects might contribute to positively modulate the immune system during stress events, including surgery.

**Fig. 2:**
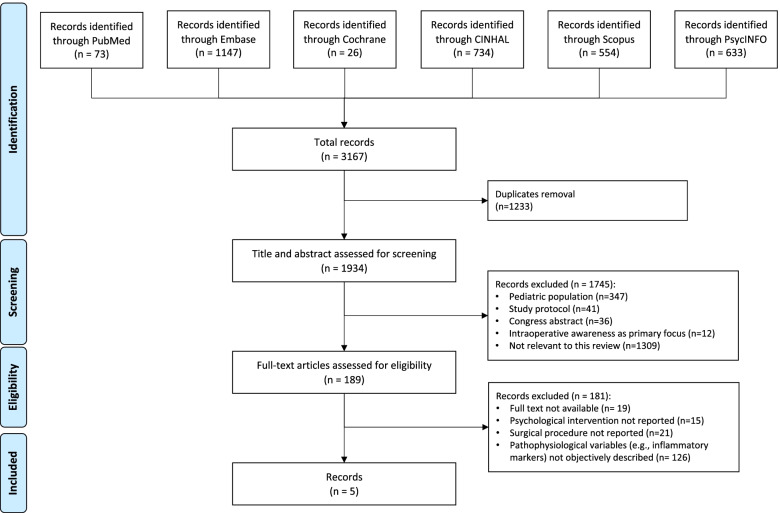
Preoperative, intraoperative, and postoperative variables influencing the patient surgical stress response. This review specifically focuses on the effects of psychological optimization (i.e., a preoperative non-pharmacological variable) in reducing the surgical stress response

Nowadays, comprehensive multimodal and multidisciplinary strategies have been developed to control the surgical stress response, reduce postoperative complications, “Enhance recovery after surgery”, and improve patients’ quality of life in the short- and long-term [27] (Fig. 2).

### Psychological treatments, surgical stress, and outcomes

**Fig. 3:**
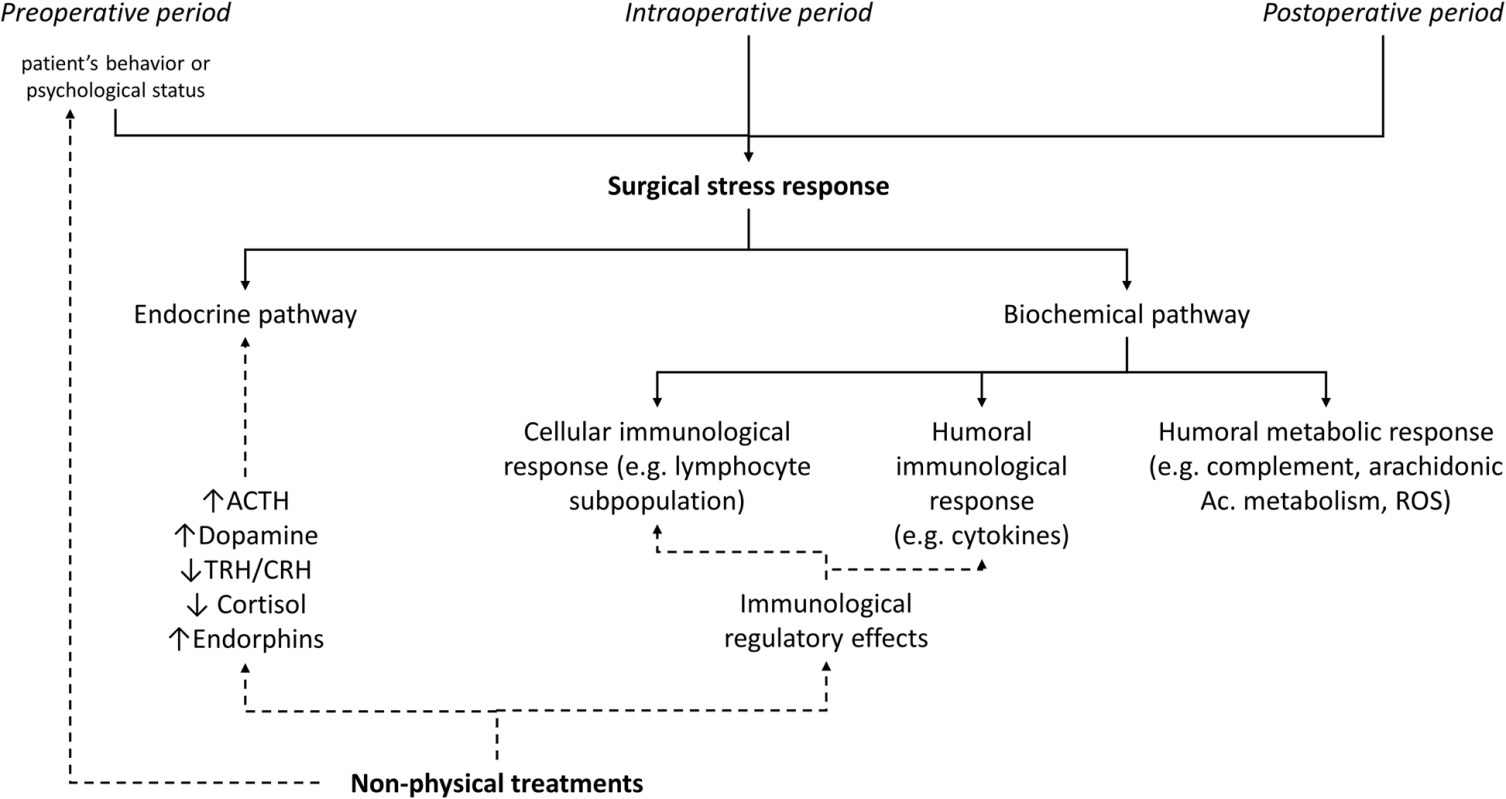
Effects of non-physical treatments on the surgical stress response

Psychological therapies include a wide range of interventions and approaches, such as cognitive behavioral therapy and narrative medicine, aimed at facilitating the mind’s capacity to influence physical health [28]. These treatments, used during psychotherapy or clinical psychology, might positively impact on the patient’s perioperative perception of emotions, cognitions, and behaviors, thus influencing surgical outcomes [29] (Fig. 3). Through the management of physical or emotional distress, psychological treatments have proved effective in reducing pharmacological treatment requirements, length of stay in hospitals, and perioperative symptoms such as pain and anxiety [30].

Studies considered in this systematic review seem to confirm that psychological strategies improve some surgical outcomes, via a positive interaction with the metabolic surgical stress response.

It is well known that the inflammatory state can be successfully modified by psychological interventions perioperatively. Indeed, in a randomized controlled trial with obese patients undergoing knee arthroplasty, Huebner et al. demonstrated a reduction in osteoarthritis-related inflammatory markers in patients receiving a 24-week cognitive behavioral intervention [31]. Similarly, as part of a randomized clinical trial, Thornton et al. randomized 45 patients with recent diagnosis of breast cancer and clinically significant depressive symptoms to receive psychological interventions [9]. The authors found a significant reduction in depressive symptoms, pain, fatigue, and improvement in markers of systemic inflammation (e.g., CD3+, CD4+, CD8+, CD56+ lymphocytes, neutrophil count, and the helper suppressor ratio CD4+/CD8+T cells). Interestingly, they also showed reduced depressive symptoms to be a consequence of intervention-related immune changes. The authors concluded that psychological interventions directly reduced inflammation and pain/depressive symptoms [9]. Currently, the effects that psychological interventions have on the neuroendocrine and inflammatory responses to surgical stress are consistently described in several observational or interventional studies [32–34]. In all studies selected in this systematic review and reported in Table 1, these biochemical effects are associated with improved surgical outcomes, such as pain and/or anxiety.

In general, cognitive-behavioral interventions and narrative medicine have both been identified as effective preoperative approaches capable of improving surgical stress response and patient outcomes. Such interventions can realistically be adopted in perioperative care and surgical procedures and should be considered a feasible option to improve clinical practice [35].

#### Cognitive behavioral therapy (CBT)

Fear-avoidance beliefs, catastrophic thinking, feelings of helplessness, and lack of control seem to be associated with passive coping strategies such as resting and avoidance behaviors. According to the fear-avoidance model, catastrophic thinking is a typical prerequisite [36]. A model based on a perception imbued with catastrophic interpretations will be based on negative beliefs and will induce patients towards avoidance behavior, thus gradually generating deconditioning and disablement. As a result, patients will deprive themselves of an increasing range of movements and activities, replacing them more with rest. These maladaptive coping strategies may delay, or even hinder, rehabilitation after surgery and increase the rates of postoperative complications. In the course of time, patients may become increasingly more disabled and limited in their work and social life, with consequent impairment of quality of life.

At the general level, the goal of cognitive behavioral therapy (CBT) is to identify and challenge maladaptive thoughts by positively modifying feelings and behaviors. The biopsychosocial approach of CBT in the medical context focuses on the complex interplay of cognitive, emotional, behavioral, and social factors, and how they interact with biomedical factors. The main assumption is that characteristics such as preoperative anxiety, fear-avoidance beliefs, maladaptive coping strategies, and pain catastrophizing may predict more severe pain, frequent postoperative complications, reduced function, and poorer quality of life after surgery [37]. Several studies involving CBT approaches aimed at targeting catastrophizing and fear avoidance behaviors have been a.ssociated with reductions in depression, physical disability, and postoperative complications [38–43]. Besides having positive effects on postoperative behaviors, moods, pain, and physical rehabilitation, CBT has been associated in the literature with reduced postoperative complications through direct interaction with the neuroendocrine pathways typically observed during maladaptive surgical stress response and described in the previous section. Some examples are described below.

#### Relaxation interventions

Relaxation techniques include physical and cognitive treatments (such as progressive muscle relaxation, simple relaxation, and breathing practices) aimed at reducing sympathetic arousal, increasing the feeling of calm, and improving control of postoperative pain [44]. In a prospective randomized controlled trial, La Montagne et al. demonstrated a positive effect of these interventions on adolescents undergoing major orthopedic surgery; in particular, relaxation therapies were statistically correlated with reduction in anxiety, pain, and postoperative complications [44]. Similar results were obtained from a more recent randomized controlled trial showing that relaxation therapy, in addition to analgesic, was effective in reducing postoperative pain without adding side effects [45]. In a study by Andersen et al., patients treated for breast cancer received relaxation techniques to reduce stress together with interventions aimed at improving mood, altering unhealthy behaviors, and maintaining adherence to cancer treatment. Patients in the interventions group showed a significant reduction in anxiety compared to the control group. Interestingly, the immune responses of patients in the interventions group were consistent with psychological and behavioral improvements; in particular, the in vitro stimulation of T cell proliferation increased in these patients [46].

Similar results were obtained by Marques et al. in a study on patients undergoing videolaparoscopic bariatric surgery. Among patients treated with relaxation therapy in this study, a significant reduction in cortisol level and anxiety symptoms was observed [47].

#### Mindfulness-based interventions (MBI)

MBIs can be seen as psychological intervention techniques inspired by religion-based practices of meditation and contemplation, nowadays rapidly emerging as effective tools in health care settings. Like other psychological interventions, MBIs promote reduction in the physical and psychological symptoms of stress [48]. MBIs presuppose patient engagement in the relevant aspects of the present experience in a non-judgmental manner: the patient is trained to suspend judgment and to divert explicit attention from a priori beliefs and other regulative representations to fully experience the present inner responses to contingency and emotions. This attitude supports the development of a greater feeling of emotional balance and well-being [49]. Mindfulness usually requires a systematic mindfulness-based stress reduction (MBSR) program that comprehends sitting meditation, group discussions, didactics, and home practice on topics including perceptions and reactions to events in life [50, 51]. MBSR has been demonstrated to be effective for improving long-term conditions including pain, anxiety, and other psychological symptoms [52].

#### Written emotional disclosure

Emotional disclosure is a psychological method that encourages patients to write, in as much detail as possible, about their feelings and emotions concerning stress experiences and/or previous traumatic events [53]. Similar to other preoperative CBT interventions, written emotional disclosure may be used to reduce stress and enhance physical and psychological health in the perioperative period, thus improving surgical outcomes and reducing hospitalization [54].

Disclosure of traumatic experiences was statistically correlated in previous studies with reduction in postoperative complications, mainly through upregulation of the immune function and more effective wound healing [55]. In a prospective controlled study, Weinman et al. investigated the impact of disclosure intervention on the progress of wound healing after punch biopsy. Patients enrolled in the experimental (emotional disclosure) group had to prepare a written report on previous traumatic and distressing experiences, paying particular attention to emotions and feelings related to those events. On the other hand, patients enrolled in the control group were asked to write about time management, trying to be as objective as possible, paying attention to details, and neglecting emotions. The authors observed that participants in the experimental group experienced a significant reduction in postoperative complications and more rapid wound healing than those in the control group [55].

#### Narrative medicine

Narrative medicine can be defined as a medical approach acknowledging the value of people’s narratives and individual stories, focusing on the relational and psychological dimensions that are implied in physical illness. According to Lewis, “even the most rigorous medical science contains human perspectives, interests, and goals imbedded in the way knowledge is selected, organized and prioritized” [48]. Over the last two decades, clinical practice enriched by narrative competence has been largely adopted as a model for humane and effective medical practice [56].

The narrative aspect of medicine had already been recognized by Hippocrates, who wrote that “the sort of disease a person has is much less important than the sort of person that has the disease.”

Indeed, one of the primary ways that humans encounter themselves and each other and deal with illness and suffering is through storytelling, that is, the process of framing one’s experience as a narrative and imbuing it with meaning. Therefore, in order to effectively help patients, healthcare professionals must participate in this process and experience the story of their illness by connecting with them personally and meaningfully [57]. Surgical patients are neither their symptoms nor their diagnoses: patients are persons who face their disease with expectations, fears, and hopes. The same principle holds true for any phase of illness and care. In conclusion, it is plausible to hypothesize that a narrative approach could enhance stress resilience in patients in the perioperative period. Nonetheless, at present—and to our knowledge—no studies are available in the literature that evaluate the direct impact of a narrative medicine approach on surgical outcomes and surgical stress response. Regarding interventions based on narrative medicine, it is desirable to suggest a potential intervention design for the future following the review by Fioretti et al. published in 2016 [58].

This review, compared to the one conducted in 2020, which defined only the effectiveness of therapies on pain and anxiety [2], has a slightly different focus in which the pathophysiological aspect was stressed more, going to show how these types of interventions may be more directly associated with the metabolic response to stress. In this regard, new articles of relevance to the field have been added and there has been a time update as well as an update on dates.

## Limitations

Several limitations can be recognized in this systematic review. First, the studies considered for the final analysis have explored different biohumoral parameters (including cortisol, cytokines, or other inflammatory mediators). A final conclusion on specific pathphysiological mechanisms or pathways cannot be made. Second, we have limited the surgical outcomes to pain and anxiety, whereas it would have been interesting to evaluate parameters such as postoperative sepsis and/or surgical site infections. Finally, we have limited the review to those psychological interventions that we consider as the most suitable for the perioperative period.

## Conclusions

Psychological characteristics can have a profound influence on maladaptive biochemical and neuroendocrine responses to surgical stress, thus potentially affecting perioperative outcomes. Similarly, psychological therapies aimed at modulating the patient’s perioperative experiences might interact with physiological responses to stress and influence surgical outcomes. Our findings confirm that psychological interventions may contribute to improve surgical outcomes via the positive influence on patients’ metabolic surgical stress response. Although meta-analyses and randomized controlled trials aimed at demonstrating the influence of psychological interventions on surgical outcomes include studies adopting several different methodologies, small to large effects were achieved, depending on the type of intervention and measured outcome. Physical therapies (e.g., anesthetic and/or surgical procedures) and non-physical therapies (e.g., cognitive behavioral therapies or narrative medicine approaches) may improve surgical outcomes, suggesting that a multidisciplinary approach, including these practices in the preoperative period, should be considered.

## Supplementary Information

**Additional file 1.**

## Data Availability

Not applicable.

## Abbreviations

SIRS: Systemic inflammatory response syndrome
TNF: Tumor necrosis factor
IL: Interleukin
CBT: Cognitive behavioral therapy
MBI: Mindfulness-based interventions
MBSR: Mindfulness-based stress reduction
