# Supplementary material for: The influence of psychological interventions on surgical outcomes: a systematic review
Source: J Anesth Analg Crit Care. 2022 Jul 9;2:31. doi: 10.1186/s44158-022-00057-4 (PMC10245433; doi:10.1186/s44158-022-00057-4)
Supplement: Supplementary file 1 — Additional file 1. [file 44158_2022_57_MOESM1_ESM.docx]

**PUBMED**

("cognitive behavioral therapy" OR "relaxation therapy" OR "mindfulness" OR "coping" OR "hypnosis", "narrative medicine" OR "psychological intervention") AND (surgery) AND (anxiety OR pain) Filters: Case Reports, Clinical Trial, Meta-Analysis, Observational Study, Randomized Controlled Trial, Review, Systematic Review, English, from 2000 – 2023.

**EMBASE**

('cognitive behavioral therapy' OR 'relaxation therapy' OR 'mindfulness' OR 'coping' OR 'hypnosis' OR 'narrative medicine' OR 'psychological intervention') AND ('surgery'/exp OR surgery) AND (‘anxiety’/exp OR anxiety OR ‘pain’/exp OR pain) ([cochrane review]/lim OR [systematic review]/lim OR [meta-analysis]/lim OR [randomized controlled trial]/lim OR 'observational study' OR 'case study') AND [english]/lim AND [2000-2022]/py.

**CINAHL**

("cognitive behavioral therapy" OR "relaxation therapy" OR "mindfulness" OR "coping" OR "hypnosis", "narrative medicine" OR "psychological intervention") AND (surgery) AND (anxiety OR pain) Limiters - Published Date: 20000101-20221231 Expanders - Apply equivalent subjects Narrow by Language: - English Search modes - Boolean/Phrase.

**COCHRANE**

(("cognitive behavioral therapy" OR "relaxation therapy" OR "mindfulness" OR "coping" OR "hypnosis", "narrative medicine" OR "psychological intervention") AND (surgery)) AND (anxiety OR pain): ti, ab, kw.

**SCOPUS**

( ( TITLE-ABS-KEY ( "cognitive behavioral therapy" OR "relaxation therapy" OR "mindfulness" OR "coping" OR "hypnosis" OR "narrative medicine" OR "psychological intervention" ) AND TITLE-ABS-KEY ( surgery ) ) ) AND ( ( anxiety OR pain ) ) AND ( TITLE-ABS ( {systematic review} OR {case series} OR {randomized controlled trial} OR rct OR {meta-analysis} OR metanalysis OR {observational study} ) ) AND ( LIMIT-TO ( LANGUAGE , "English" ) ) AND ( LIMIT-TO ( PUBYEAR , 2021 ) OR LIMIT-TO ( PUBYEAR , 2020 ) OR LIMIT-TO ( PUBYEAR , 2019 ) OR LIMIT-TO ( PUBYEAR , 2018 ) OR LIMIT-TO ( PUBYEAR , 2017 ) OR LIMIT-TO ( PUBYEAR , 2016 ) OR LIMIT-TO ( PUBYEAR , 2015 ) OR LIMIT-TO ( PUBYEAR , 2014 ) OR LIMIT-TO ( PUBYEAR , 2013 ) OR LIMIT-TO ( PUBYEAR , 2012 ) OR LIMIT-TO ( PUBYEAR , 2011 ) OR LIMIT-TO ( PUBYEAR , 2010 ) OR LIMIT-TO ( PUBYEAR , 2008 ) OR LIMIT-TO ( PUBYEAR , 2007 ) OR LIMIT-TO ( PUBYEAR , 2005 ) OR LIMIT-TO ( PUBYEAR , 2001 ) OR LIMIT-TO ( PUBYEAR , 2000 ) )

**PsycHINFO**

("cognitive behavioral therapy" OR "relaxation therapy" OR "mindfulness" OR "coping" OR "hypnosis", "narrative medicine" OR "psychological intervention") AND (surgery) AND (anxiety OR pain) Limiters - Published Date: 20000101-20221231 Expanders - Apply equivalent subjects Narrow by Language: - English Search modes - Boolean/Phrase.
